# Professionals' Perceptions of the Management of Digital Competence Sharing in Healthcare and Associated Background Factors: A Cross‐Sectional Study

**DOI:** 10.1111/jan.17055

**Published:** 2025-05-10

**Authors:** Mira Hammarén, Tarja Pölkki, Outi Kanste

**Affiliations:** ^1^ Research Unit of Health Sciences and Technology University of Oulu Oulu Finland; ^2^ Research Unit of Health Sciences and Technology, Medical Research Center Oulu University Hospital and University of Oulu Oulu Finland

**Keywords:** digital competence, healthcare, knowledge sharing, management, survey

## Abstract

**Aims:**

To describe professionals' perceptions of the management of digital competence sharing in healthcare and associated background factors.

**Design:**

A descriptive cross‐sectional study.

**Methods:**

The study used an online survey involving 227 healthcare professionals from three public and one private healthcare organisation in Finland. Data was collected using the management of digital competence sharing (MDCS) instrument and analysed using descriptive statistics, independent sample *t*‐tests and one‐way ANOVA.

**Results:**

Based on the professionals' perceptions, the overall management of digital competence sharing was weak. They perceived the highest level of creation of a friendly and safe digital organisational atmosphere while the lowest level of provision of resources and opportunities for digital competence sharing. Background factors, including gender, age, work experience in healthcare, organisation and clinical environment, showed statistically significant differences in how professionals perceived the management of digital competence sharing.

**Conclusion:**

The results emphasised the need for increased managers' attention to digital competence development, prioritising and supporting digital competence sharing among healthcare professionals.

**Implications:**

The results can be utilised in healthcare management to enhance the digital competence sharing among healthcare professionals and the use of existing digital competence to benefit the work community.

**Impact:**

The importance of digital competence is increasing among healthcare professionals, but at the same time, they perceive inadequate management support in this area. This study revealed limited management of digital competence sharing in healthcare organisations, particularly among older professionals and those in inpatient and primary care settings. These results can be applied in managers' training to support and promote digital competence among healthcare professionals.

**Reporting Method:**

The STrengthening the Reporting of OBservational studies in Epidemiology (STROBE) checklist.

**Patient or Public Contribution:**

There is no patient or public contribution.

**Summary**

- What does this paper contribute to the wider global clinical community?
  ○ This study provides new insights into professionals' perceptions of the management of digital competence sharing, which have not previously been studied in a large population.
  ○ The results can help management support digital competence sharing among professionals, improving a culture of knowledge sharing.
  ○ The results can be applied to strengthen professionals' digital competence and in training managers.

## 1 Introduction

The growing use of digital technologies is having a significant impact on the management of healthcare systems (Dal Mas et al. 2023). This technological advancement has underscored the importance of understanding digital competence in healthcare (Alhur et al. 2023). The European Parliament and the Council identified digital competence as one of eight critical competencies for lifelong learning (European Commission: Directorate‐General for Education, Youth, Sport and Culture 2019). The digital competence of professionals is essential for improving patient care (Jarva et al. 2024) and safety by enabling the effective use of advanced technologies (Alhur et al. 2023). Digital competence among healthcare professionals correlates with better patient outcomes, increased patient satisfaction, and higher patient engagement levels (Alhur et al. 2023). Better digital competence of healthcare professionals enhances efficiency and productivity through better use of digital health solutions (Dal Mas et al. 2023), promoting the adoption of digital solutions (Jarva et al. 2024). However, effective utilisation of digital solutions by healthcare professionals necessitates an understanding of their potential benefits and continuous competence development (Popov et al. 2022).

Healthcare organisations must address the needs of healthcare professionals by providing support and training and considering potential workload reductions (World Health Organization [WHO] 2023). However, the training and support provided by organisations to cope with digital transformation are inadequate (Kaihlanen et al. 2024; Navarro‐Martínez et al. 2023). High levels of digital competence among professionals have been positively associated with job satisfaction and workability. Conversely, technology‐related stress has significantly weakened workability and increased the intention to leave the organisation (Golz et al. 2021). Therefore, managers must ensure that healthcare personnel are adequately prepared and supported during the transition to new digital solutions (Dal Mas et al. 2023). Sharing digital competence between professionals is one way to promote utilising existing digital expertise in organisations. This continuous learning culture might help bridge the digital divide.

## 2 Background

There is no precise concept of digital competence in healthcare (Mainz et al. 2024). Still, the phenomenon has been viewed from many different angles and with varying degrees of precision, such as informatics competence (Jedwab et al. 2023) or digital skills (Kaihlanen et al. 2024). However, the definitions focus on the interaction between people and technology and how this supports learning, working and knowledge sharing (Clarke‐Darrington et al. 2023). Digital competence can be broadly seen as a combination of knowledge, skills, and attitudes, motivation, and individual characteristics related to digital technologies (Ochoa Pacheco and Coello‐Montecel 2023). It describes an individual's ability to communicate on digital platforms, search and evaluate digital knowledge, create digital content and solve technological problems (Ochoa Pacheco and Coello‐Montecel 2023; Vuorikari et al. 2022). In healthcare, digital competence involves basic computer and technology skills, understanding and using digital tools in patient care, analysing healthcare data, and managing electronic documentation (Jimenez et al. 2020). In addition, it includes the ability to provide e‐health services (Longhini et al. 2022; Mainz et al. 2024), communicate via tele‐technology, and make ethical decisions (Jarva et al. 2024; Jimenez et al. 2020).

Knowledge sharing is significant in knowledge management and linked to organisational efficiency (Pandey et al. 2021). Management of digital competence sharing requires knowledge management. By assessing and recognising healthcare professionals' digital competence strengths, managers can leverage these competencies (Hammarén et al. 2023). Knowledge sharing involves exchanging expertise, providing feedback or sharing information (Kim and Park 2017), which can occur in both formal and informal settings (Sabeeh et al. 2018). In this study, digital competence sharing refers to sharing digital information, skills, and related attitudes among healthcare professionals in organisations. This knowledge sharing can occur informally through interaction, such as advising others during work or discussions, and formally through methods like giving instructions, mentoring, or assigning responsibilities (Hammarén et al. 2023).

The management of digital competence encompasses managers' actions to support and enhance digital competence sharing, such as creating a friendly and safe atmosphere, creating methods and practices, utilising and recognising digital competence, providing resources for digital competence sharing, and supporting digital competence sharing through leadership (Hammarén et al. 2023). Developing digital competency for professionals involves strategic planning and creating processes and structures that foster a supportive environment for digital competence (Chen et al. 2022). This underscores the importance of creating a friendly and safe digital organisational atmosphere, where managers actively foster a culture that values and respects digital competence (Hammarén et al. 2023) In healthcare settings, such an atmosphere encourages professionals to engage in digital development and share their knowledge without fear of judgement (Sabeeh et al. 2018). Creating methods and practices for digital competence sharing involves establishing structured ways to promote collaboration and mutual learning (Hammarén et al. 2023). From a management perspective, identifying and utilising professionals' digital competence involves recognising digital strengths across individuals and allocating tasks accordingly, which supports tailored development and promotes mentorship among peers (De Leeuw et al. 2020; Hammarén et al. 2023). Providing resources and opportunities for digital competence sharing is grounded in the recognition that a lack of time, support, and opportunities are key barriers to digital development in healthcare (Hayward and Dube 2023). To overcome this, managers must ensure time allocation, balanced workloads, and access to development opportunities such as workshops and collaborative learning platforms (Turulja et al. 2021). Leadership in promoting digital competence sharing involves managers' characteristics, such as positive attitudes and openness towards digitalisation (Hammarén et al. 2023).

One of the barriers to knowledge sharing in organisations is a lack of practice and arrangement for knowledge sharing (Al Mashmoum and Hamade 2019). Previous studies have highlighted that professionals often face barriers such as lack of time, insufficient training and knowledge of digital competence (De Leeuw et al. 2020; Hayward and Dube 2023). To address these barriers, it has been suggested that opportunities to develop digital competence, such as seminars, workshops and educational programmes, should be offered (Turulja et al. 2021). Recognising that not all professionals must be digital experts is important because having a small number of digital experts in the workforce ensures that others can access current training (Morris et al. 2023).

Previous studies have examined the digital competence of healthcare professionals (Jarva et al. 2024) and managers (Jedwab et al. 2023). Additionally, studies have explored knowledge sharing and its related factors, such as the utilisation of web platforms for knowledge sharing (Maheshwari et al. 2021). In the healthcare field, knowledge sharing has been studied, especially regarding experienced professionals sharing their knowledge with younger ones, and different governance mechanisms have been developed for this purpose (Tabrizi and Morgan 2014). However, there is limited evidence on the management of digital competence sharing. This study provides new insights into how digital competence sharing is supported in healthcare organisations and which background factors are associated with it. It is important to explore the association between background factors and the management of digital competence sharing, as younger age has been found to be associated with higher levels of digital competence (Golz et al. 2021) and differences in the provision of digital competence training have been observed between healthcare organisations (Navarro‐Martínez et al. 2023). With these results, management can effectively promote the digital competence of healthcare professionals, thereby enhancing healthcare services.

## 3 The Study

### 3.1 Aim

This study aimed to describe professionals' perceptions of the management of digital competence sharing in healthcare and associated background factors.

This study addressed the following research questions:

1. How do professionals perceive the management of digital competence sharing in healthcare?
2. Which background factors are associated with the management of digital competence sharing in healthcare?

## 4 Methods

### 4.1 Study Design

The study was conducted as a descriptive cross‐sectional study using an online survey. The STROBE checklist for cross‐sectional studies was used in reporting (von Elm et al. 2007).

### 4.2 Study Settings and Sample

Participants encompassed healthcare professionals from three public and one private healthcare organisation in Finland, including four hospitals. In Finland, public healthcare organisations provide health and social services that cover primary and specialised care. The private healthcare organisation operated one hospital providing special healthcare services. Healthcare organisations in Finland extensively use digital health services and technological solutions, including digital care pathways, virtual hospital systems, remote clinics, robotics and other technological innovations, making the integration of digital solutions and technology an integral part of every professional's work. The inclusion criteria for professionals were a professional degree in health care (e.g., registered nurse, midwifery, practical nurse, physiotherapist), regardless of role, responsibilities, or involvement in digital competence sharing. A convenience sample was used to select participants (Polit and Beck 2018). The target organisation was chosen from health services for older people, primary healthcare, and specialist healthcare services, covering outpatient and inpatient services. These units employed a diverse range of healthcare professionals and had a substantial number of staff members.

### 4.3 Data Collection

Data was collected using an online survey between August and October 2024. The power analysis, using G*power, was conducted using a previous study (Wagner et al. 2019) to estimate the sample size needed. Power analysis was performed assuming significance level *α* = 0.05 and power of 0.8 (*β* = 0.2), and an effect size of 0.377, resulting in a sample of at least 176 healthcare professionals needed. To ensure that this sample is representative of the larger population of healthcare professionals, the demographics of the sample were compared to the demographics of the target population. According to the 2024 statistics, approximately 400,000 social and healthcare professionals are working in Finland. Of these, about 85% are women and 15% are men, with over 65% working in public healthcare organisations.

To gather a wide range of perspectives from various organisations and clinical settings, and considering the potentially low response rate, the survey was sent to professionals (*n* = 4500) through organisations' contact persons. They directed the survey invitation to the front‐line managers of the selected units, who distributed it to healthcare professionals via email in their respective units. Participants had 1 month to respond, with two reminders sent at 2‐week intervals (Polit and Beck 2018). A total of 248 healthcare professionals responded to the survey, resulting in a response rate of 5.5%.

Before analysis, the data were reviewed with regard to the ‘cannot say’ response option. Since this option could not be assigned a specific value, it was coded as a missing value. Twenty‐one participants who selected ‘cannot say’ for more than half of the items were excluded from the analysis. Consequently, responses from 227 healthcare professionals were included in the final data.

### 4.4 Instrument

The management of digital competence sharing (MDCS) instrument for healthcare professionals was used in this study. The MDCS instrument consists of five sum variables and a total of 34 items: (1) creating a friendly and safe digital organisational atmosphere (eight items), (2) creating methods and practices of digital competence sharing (eight items), (3) identifying and utilising professionals' digital competence (seven items), (4) providing resources and opportunities for digital competence sharing (five items) and (5) leadership in promoting digital competence sharing (six items). In the instrument, participants assess the actions taken by their frontline manager related to the management of digital competence sharing using a five‐point Likert scale (1 = completely disagree, 2 = partially disagree, 3 = partially agree, 4 = completely agree and 5 = cannot say).

The instrument was developed by the research group and is based on a qualitative interview study that describes healthcare managers' and professionals' perceptions of the management of digital competence sharing (Hammarén et al. 2023). The content validity of the instrument was evaluated by an expert panel (*n* = 8), comprising researchers, healthcare professionals and managers with experience in digital healthcare and management. A pilot study was conducted with four healthcare professionals and four managers to determine the response time, assess the instrument's usability, and ensure that the target group understood the items. The structural validity of the instrument was assessed using exploratory factor analysis, resulting in five sum variables. Cronbach's alpha values were calculated for each sum variable to assess the instrument's internal consistency, ranging from 0.91 to 0.95 (Table 1). In addition, participants were asked about background factors: gender, age, work experience, professional background, clinical working environment, organisation and education. Participants were required to answer all items on the instrument, as well as the background questions.

**TABLE 1:** Sum variables of the management of digital competence sharing (MDCS) instrument.

Sum variable of MDCS instrument	Number of items	Description	Cronbach's alpha
Creating a friendly and safe digital organisational atmosphere	8	A manager promotes a supportive and safe digital atmosphere by respecting and valuing digital competence, encouraging their development, and effectively communicating the benefits and changes associated with digitalisation	0.949
Creating methods and practices for digital competence sharing	8	A manager creates methods and practices for sharing digital competence by encouraging collaboration and mutual support, creating guidelines, and facilitating teamwork and training	0.912
Identifying and utilising professionals' digital competence	7	A manager identifies and exploits the digital strengths of different professionals and generations, allocates tasks based on digital competence and guides people to mentor others	0.937
Providing resources and opportunities for digital competence sharing	5	A manager allocates time and organises working conditions to facilitate digital competence sharing, ensuring equal consideration of workload and providing opportunities for collaborative learning	0.952
Leadership in promoting digital competence sharing	6	A manager promotes digital competence sharing through leadership by demonstrating digital competence, ensuring professionals have the necessary competence, anticipating competence needs and identifying barriers	0.911

### 4.5 Data Analysis

Participant background factors were described using frequencies, percentages (%), means and standard deviations (SD). Professionals' perceptions of the management of digital competence sharing in healthcare were described in five sum variables and presented in terms of means and standard deviations. The means of the sum variables were categorised into three levels (weak = 1–2.499, good = 2.5–3.499 and very good = 3.5–5) and described using percentages (%). The association between the participant's background factors and sum variables was analysed using the independent samples *t*‐test and one‐way analysis of variance (ANOVA), with statistical significance identified at *p* < 0.05. After detecting statistically significant differences, Tukey's HSD (honestly significant difference) test was applied to assess how each group differed significantly from the others. The data were analysed using the statistical software IBM SPSS Statistics for Windows (version 29.0).

### 4.6 Ethical Considerations

Research permits were applied from target organisations. There was no need to request a prior assessment by the Ethics Committee because the study did not concern patients or minors and did not intervene with the physical or mental integrity of the participants (World Medical Association 2013). Study participants were provided with written information detailing the study's aims, contact information, implementation of the study, voluntary participation and the possibility to suspend participation at any time. At the beginning of the survey, participants gave informed consent to use the collected data for research purposes. They were also informed that their anonymity would be fully protected and could not be identified based on the study results. The study collected only the personal data necessary for the study. Collecting and processing personal data adhered to the European Union's data protection legislation (European Union 2016). The data will be discarded after the results have been published at the end of the study.

## 5 Results

### 5.1 Participants' Background Factors

Of the 227 healthcare professionals, the majority (89.0%) were female, with a mean age of 45.3 years. Over half of the participants (56.8%) were registered nurses, followed by practical nurses, who comprised 18.9%. Additionally, more than half (59.5%) of the participants worked in public specialised healthcare (Table 2).

**TABLE 2:** Participants and background factors (*n* = 227).

Background factors	*n*	%	Mean (SD)	Median (range)
Gender				
Female	202	89.0		
Male	19	8.4		
Prefer not to say	6	2.6		
Age			36.3 (11.6)	35.0 (49)
< 30	15	6.6		
30–39	55	24.2		
40–49	70	30.8		
≥ 50	87	38.3		
Work experience in healthcare			16.9 (10.7)	16.9 (45)
< 5 years	32	14.1		
5–14	64	28.2		
≥ 15	131	57.7		
Professional background				
Registered nurse	129	56.8		
Practical nurse	43	18.9		
Physical therapist	18	7.9		
Midwife	6	2.6		
Dental hygienist/nurse	6	2.6		
Public health nurse	5	2.2		
Speech therapist	4	1.8		
Occupational therapist	3	1.3		
Other^a^	13	5.7		
Organisation				
Public specialised healthcare	135	59.5		
Public primary healthcare	64	28.2		
Private healthcare	28	12.3		
Clinical working environment				
Inpatient^b^	117	51.5		
Outpatient^c^	110	48.5		
Educational level				
Second‐level degree or lower	97	42.7		
Bachelor's degree	97	42.7		
Master's degree or higher	33	14.5		

^a^ Radiographer, psychologist, audiologist, pharmacist and nutritionist.

^b^ Hospital ward, intensive care unit, operating theatre, delivery room and nursing home.

^c^ Outpatients clinics, day surgery unit, home care, assisted living, rehabilitation services, dental care and laboratory.

### 5.2 Healthcare Professionals' Perceptions of the Management of Digital Competence Sharing

The sum variable for creating a friendly and safe digital organisational atmosphere had the highest mean of 2.769 (SD 0.797) (Table 3). This was perceived as weak by 17.6% of participants, good by 35.2% and very good by 11.9%. It was also the sum variable most frequently answered as ‘cannot say’ (35.2%). Leadership in promoting digital competence sharing received the second highest mean of 2.532 (SD = 0.728), followed by the creation methods and practices for digital competence sharing, with a mean of 2.419 (SD = 0.797).

**TABLE 3:** Healthcare professionals' perceptions of the management of digital competence sharing (*n* = 227).

Sum variable of management of digital competence sharing	*n*	Mean (SD)	Weak^a^	Good^b^	Very good^c^	Cannot say^d^
			% (*n*)	% (*n*)	% (*n*)	% (*n*)
Creating a friendly and safe digital organisational atmosphere	147	2.769 (0.797)	17.6 (40)	35.2 (80)	11.9 (27)	35.2 (80)
Creating methods and practices for digital competence sharing	181	2.419 (0.797)	37.4 (85)	36.1 (82)	6.2 (14)	20.3 (46)
Identifying and utilising professionals' digital competence	156	2.351 (0.862)	36.6 (83)	25.6 (58)	6.6 (15)	31.3 (71)
Providing resources and opportunities for digital competence sharing	185	2.124 (0.728)	57.3 (130)	22.5 (51)	1.8 (4)	18.5 (42)
Leadership in promoting digital competence sharing	154	2.532 (0.778)	26.4 (60)	33.5 (76)	7.9 (18)	32.2 (73)

^a^ Weak = 1–2.499.

^b^ Good = 2.5–3.499.

^c^ Very good = 3.5–4.

^d^ Cannot say = 0.

The sum variable for providing resources and opportunities for digital competence sharing received the lowest mean of 2.124 (SD = 0.728). This was perceived as weak by 57.3% of participants, good by 22.5% and very good by 1.8%. However, it had the lowest percentage of ‘cannot say’ responses (18.5%). The second lowest mean was for identifying and utilising professionals' digital competence, with a mean of 2.351 (SD = 0.862).

### 5.3 The Background Factors Associated With the Management of Digital Competence Sharing

Among background factors, gender, age, and work experience in healthcare, organisation, and clinical environment showed statistically significant differences in perceptions of the management of digital competence sharing (Table 4). Males perceived the identification and utilisation of digital competence as lower than females. Regarding age, those under 30 years old perceived the creation methods and practices for digital competence sharing higher than those aged 40–49 years (*p* = 0.037) and those over 50 years (*p* = 0.042). Additionally, they perceived the identification and utilisation of professionals' digital competence higher than those 40–49 years old (*p* = 0.011). However, professionals with 5–14 years of work experience in healthcare perceived the creation methods and practices for digital competence sharing higher than those with over 15 years of work experience (*p* = 0.034).

**TABLE 4:** The association between the background factors and the management of digital competence sharing in healthcare (*n* = 227).

Background factors	Creating a friendly and safe digital atmosphere		Creating methods and practices		Identifying and utilising digital competence		Providing resources and opportunities		Leadership in promoting digital competence sharing	
	Mean (SD)	*p*	Mean (SD)	*p*	Mean (SD)	*p*	Mean (SD)	*p*	Mean (SD)	*p*
Gender.										
Female	2.798 (0.797)	0.236^a^	2.449 (0.776)	0.433^a^	2.412 (0.856)	**0.017** ^a^	2.161 (0.726)	0.196^a^	2.562 (0.774)	0.530^a^
Male	2.453 (0.790)		2.283 (0.809)		1.797 (0.745)		1.892 (0.641)		2.416 (0.705)	
Age										
< 30	3.042 (0.550)	0.361^b^	3.000 (0.323)	**< 0.001** ^b^	3.086 (0.427)	**< 0.001** ^b^	2.600 (0.638)	0.084^b^	2.803 (0.510)	0.436^b^
30–39	2.857 (0.831)		2.597 (0.814)		2.373 (0.947)		2.017 (0.664)		2.607 (0.819)	
40–49	2.628 (0.756)		2.287 (0.714)		2.162 (0.763)		2.117 (0.715)		2.418 (0.779)	
≥ 50	2.748 (0.841)		2.309 (0.811)		2.353 (0.871)		2.115 (0.778)		2.519 (0.788)	
Work experience in healthcare										
< 5 years	3.063 (0.645)	0.051^b^	2.531 (0.696)	**0.034** ^b^	2.417 (0.830)	0.590^b^	2.248 (0.738)	0.438^b^	2.698 (0.651)	0.221^b^
5–14	2.859 (0.864)		2.609 (0.830)		2.432 (0.906)		2.167 (0.703)		2.627 (0.808)	
≥ 15	2.631 (0.771)		2.286 (0.758)		2.284 (0.850)		2.067 (0.739)		2.434 (0.783)	
Professional background										
Nursing^c^	2.759 (0.810)	0.370^b^	2.427 (0.788)	0.620^b^	2.359 (0.851)	0.809^b^	2.114 (0.723)	0.852^b^	2.540 (0.768)	0.244^b^
Rehabilitation^d^	2.646 (0.516)		2.241 (0.613)		2.186 (0.777)		2.165 (0.609)		2.289 (0.725)	
Other^e^	3.161 (0.909)		2.534 (0.952)		2.429 (1.340)		2.250 (1.340)		2.833 (0.965)	
Organisation										
Public specialised healthcare	2.919 (0.843)	**0.013** ^b^	2.453 (0.892)	**0.005** ^b^	2.456 (0.931)	0.146^b^	2.215 (0.799)	**0.042** ^b^	2.763 (0.732)	**0.002** ^b^
Public primary healthcare	2.619 (0.776)		2.333 (0.752)		2.257 (0.830)		2.028 (0.694)		2.346 (0.786)	
Private healthcare	3.114 (0.666)		2.819 (0.551)		2.661 (0.826)		2.430 (0.637)		2.886 (0.601)	
Clinical working environment										
Inpatient^f^	2.672 (0.765)	0.091^a^	2.432 (0.718)	0.797^a^	2.314 (0.787)	0.512^a^	2.101 (0.668)	0.610^a^	2.412 (0.723)	**0.027** ^a^
Outpatient^g^	2.897 (0.836)		2.400 (0.873)		2.408 (0.973)		2.156 (0.808)		2.692 (0.824)	
Educational level										
Second‐level degree or lower	2.841 (0.954)	0.211^b^	2.496 (0.868)	0.235^b^	2.465 (0.915)	0.533^b^	2.248 (0.779)	0.272^b^	2.616 (0.899)	0.686^b^
Bachelor's degree	2.800 (0.705)		2.433 (0.751)		2.313 (0.844)		2.078 (0.702)		2.500 (0.737)	
Master's degree or higher	2.488 (0.694)		2.169 (0.694)		2.248 (0.831)		2.017 (0.698)		2.485 (0.676)	

*Note:* Bold values = statistical significance, *p* < 0.05.

Abbreviation: SD, standard deviation.

^a^ Independent samples *t*‐test.

^b^ One‐way analysis of variance (ANOVA).

^c^ Registered nurse, practical nurse, public health nurse, midwife and dental nurse.

^d^ Physiotherapist, occupational therapist, speech therapist and rehabilitation counsellor.

^e^ Radiographer, psychologist, audiologist, pharmacist and nutritionist.

^f^ Hospital ward, intensive care unit, operating theatre, delivery room and nursing home.

^g^ Outpatient clinics, day surgery unit, home care, assisted living, rehabilitation services, dental care and laboratory.

Regarding organisation and clinical work environment, professionals from private healthcare organisations perceived the creation of a friendly and safe digital organisational atmosphere (*p* = 0.023) and the creation of methods and practices for digital competence sharing (*p* = 0.028) higher than those from public primary healthcare organisations. Additionally, leadership in promoting digital competence sharing was perceived higher by professionals from both private healthcare (*p* = 0.018) and public specialised healthcare organisations (*p* = 0.016) compared to those from public primary healthcare organisations. Regarding the clinical work environment, professionals working in outpatient settings perceived leadership in promoting digital competence sharing as higher than those in inpatient settings. No statistically significant associations were found between professional background, education level and the sum variables.

## 6 Discussion

This study provides new insights into management's role in promoting digital competence sharing and describes managers' activities from the professionals' perspective. Previous studies have focused on how management supports digital competence among professionals through training and resources (Dugstad et al. 2020; Hayward and Dube 2023; Navarro‐Martínez et al. 2023). As digital solutions and innovations continue to advance (Alhur et al. 2023), digital competence sharing will become increasingly important. Moreover, previous studies have not examined knowledge sharing in the context of digital competence, primarily examining the phenomenon from an organisational perspective (Kim and Park 2017).

Our study investigated how professionals perceived the management of digital competence sharing, an area not previously explored. According to our results, professionals generally perceived the management of digital competence sharing in healthcare organisations as weak. This finding aligns with previous literature identifying barriers to digital competence and adopting digital systems (Dal Mas et al. 2023). Prior studies have revealed that healthcare professionals have experienced inadequate support from management in supporting digital competence (Navarro‐Martínez et al. 2023) and insufficient training opportunities (Navarro‐Martínez et al. 2023; Morris et al. 2023). Furthermore, the information provided at the beginning of digital systems implementation has been perceived as insufficient (Dugstad et al. 2020). In our study, professionals perceived the creation of a friendly and safe digital organisational atmosphere as the highest. Previous studies have identified the importance of culture creation as an integral component of supporting healthcare professionals' digital competence (De Leeuw et al. 2020; Laukka et al. 2022). In particular, managers should promote a team culture based on collegial learning where helping others is encouraged (De Leeuw et al. 2020).

In our results, professionals generally perceived the leadership in promoting digital competence sharing as good. Although leadership behaviours and characteristics related to enhancing digital competence sharing have not been previously studied, it has been recognised that manager support for digital competence has been perceived as insufficient (Navarro‐Martínez et al. 2023), and the importance of managers' role in promoting digital competence has been acknowledged (De Leeuw et al. 2020; Jarva et al. 2024). In the Jarva et al. (2024) study, management support was associated with the digital competence of professionals in integrating digital solutions into their work and in the ethical competence related to digital solutions. In addition, the manager's ability to listen to professionals' problems, understand their challenges with technology (De Leeuw et al. 2020), and provide encouragement and motivation to use technology (Laukka et al. 2022) was considered important in promoting digital competence.

Based on our results, providing recourse and opportunities for digital competence sharing was perceived as the lowest, with over half of the professionals perceiving this as weak. Previous studies have indicated that resources to enhance digital competence (Morris et al. 2023) and training were inadequate among professionals (Hayward and Dube 2023; Navarro‐Martínez et al. 2023). According to Navarro‐Martínez et al.'s (2023) study, nurses received less technology‐related training compared to physicians, and over half of both physicians and nurses have not received any technological training (Navarro‐Martínez et al. 2023). Furthermore, it has been identified that the heavy workload, stress and lack of time of professionals are barriers to digital learning at work (De Leeuw et al. 2020).

Our results highlighted that the creation methods and practices for digital competence sharing, as well as the identification and utilisation of professionals' digital competence, were perceived as weak by professionals. Previous studies have also found a lack of methods to support digital competence in healthcare organisations, and clear methods and practices to strengthen digital competence are needed (De Leeuw et al. 2020). In particular, studies have indicated the need for managers to understand and be aware of the digital competence of professionals and to identify their need for support (De Leeuw et al. 2020; Laukka et al. 2022).

Our results showed that in background factors, younger professionals perceived the creation practices and methods for digital competence sharing, as well as the identification and utilisation of professionals' digital competence, higher than older professionals. This may partly be explained by findings that younger professionals have been found to have higher levels of digital competence (Golz et al. 2021) and, therefore, less need for competence sharing. According to the study by Jarva et al. (2024), professionals with higher levels of digital competence were, on average, slightly younger and had less work experience. However, no statistically significant differences were found in these background factors concerning digital competence in their study.

A new result of this study indicated that professionals in private healthcare organisations perceived the management of digital competence sharing as the highest. This could be linked to organisational culture and resource allocation. In contrast, a study by Navarro‐Martínez et al. (2023) found no difference in the training received between primary care and hospital nurses. However, variations were noted among physicians, with those working in hospitals receiving more training than their counterparts in primary care (Navarro‐Martínez et al. 2023). In our results, professionals in outpatient settings perceived leadership in promoting digital competence sharing as higher than those in inpatient settings. Outpatient care often requires the use of digital tools for scheduling, patient communication, and data sharing across multiple care providers, possibly leading to more proactive leadership in digital competence development. Conversely, inpatient settings, where routines may be more standardised and hierarchical, could experience a slower uptake of digital innovations, as also reflected in the lower levels of digital competence reported among these professionals (Jarva et al. 2024).

Several professionals were unable to provide their perceptions on the management of digital competence sharing. This may partly reflect a single manager's increased number of subordinates in healthcare organisations, resulting in reduced direct contact between professionals and their managers. The majority of ‘cannot say’ responses were associated with the sum variable concerning creating a friendly and safe digital organisational atmosphere. The prevalence of ‘cannot say’ responses may partly indicate that management practices related to creating such an atmosphere are more challenging to observe in concrete terms. Conversely, providing resources for digital competence sharing, which professionals can concretely perceive, received the fewest ‘cannot say’ responses.

### 6.1 Limitations

The first limitation is the low response rate (5.5%) and the study's focus on Finland, which may introduce sampling bias and limit the generalisability of the results. This study aims to describe professionals' perceptions at a single point in time and does not seek to establish causality. It is also important to note that the inclusion of participants with limited relevance to the research topic may diminish the significance of the results. During the data collection, public health organisations experienced reductions in healthcare professionals due to organisational restructuring, which may have influenced the response rate. Additionally, due to differences in healthcare systems, caution should be exercised when generalising the results of this study. The second limitation is the demographic composition of the sample, as most participants were women and more than half were nurses, with no physicians included. However, in Finland and many other countries, women and nurses constitute the majority of healthcare professionals. The results of this study could be confirmed by a more representative sample, including physicians. A third limitation is that the study did not employ random sampling or ensure balanced representation across professional groups, which may have introduced sampling bias (Polit and Beck 2018).

### 6.2 Recommendations for Further Research

For further research, it is recommended to conduct a study on the management of digital competence sharing among healthcare professionals, including physicians, from a broader perspective. Since the management of digital competence sharing was perceived higher in private organisations, it is important to examine the phenomenon more broadly, including a wider range of representatives from the private healthcare sector. Additionally, the phenomenon should be studied from the perspective of healthcare managers, exploring possible differences between managers' and professionals' perceptions. Future research should also investigate educational interventions aimed at developing the competence of managers to manage digital competence sharing effectively.

## 7 Conclusion

Healthcare professionals perceived the management of digital competence sharing in healthcare organisations as limited. The creation of a friendly and safe digital organisational atmosphere and the leadership in promoting digital competence sharing were perceived as the highest and generally assessed as being at a good level. However, the provision of resources and opportunities for digital competence sharing was perceived as the lowest. This finding emphasises the need for management to further prioritise and support the development of digital competencies among healthcare professionals. Gender, age, work experience in healthcare, organisation and clinical work environment were associated with how professionals perceived the management of digital competence sharing. Among these, organisation and clinical work environment emerged as the most associated factors. Professionals working in private healthcare organisations and those in outpatient settings perceived management support as the highest. Therefore, management should take into account individual competence and support needs to effectively promote digital competence sharing.

### 7.1 Implications for Practice

Implementing digital competence sharing in healthcare organisations necessitates a comprehensive approach. Healthcare professionals must be given adequate time and opportunities to disengage from direct patient care to develop and share their digital competence. Furthermore, managers should prioritise the integration of methods that support competence sharing in everyday practice, such as mentorship programmes, peer learning, and collaborative workshops. Additionally, professionals perceive that managers do not recognise or utilise their digital competence effectively, and therefore, management should implement tools and frameworks for assessing digital competence and offer tailored training programmes based on identified needs. Creating a friendly and safe atmosphere is crucial for encouraging professionals to value mutual assistance and engage in the development of digital competence. Registered nurses constitute the largest group of healthcare professionals, and their involvement is essential. Strengthening their digital competence can positively impact the entire healthcare team and enhance patient outcomes and satisfaction. Effective nursing management is vital for facilitating education, promoting collaborative learning, and ensuring seamless teamwork among nurses.

## Author Contributions

Conceptualisation, writing, review and editing: Mira Hammarén, Tarja Pölkki and Outi Kanste. Data collection and analysis: Mira Hammarén. All authors have agreed on the final version.

## Disclosure

The statistics were checked prior to submission by an expert statistician, Hannu Vähänikkilä (hannu.vahanikkila@oulu.fi). There is no patient or public contribution.

## Conflicts of Interest

The authors declare no conflicts of interest.

## Supporting information


Data S1.

## Data Availability

Research data are not shared.
